# CD19, immunoglobulin level, and varied anti-cytokine autoantibodies underline dichotomous susceptibility to types of infection in patients with thymomas

**DOI:** 10.3389/fimmu.2026.1824198

**Published:** 2026-07-10

**Authors:** Zhaohong Tan, Areum Shin, Rachel Ying Min Tan, Dongling Wang, Chiung-Hui Huang, Sharada Ravikumar, Liang En Wee, Yvonne Fu Zi Chan, Ying Ying Chua, Sen Hee Tay, Anindita Santosa, Gladys Gek Yen Tan, Doo Ri Kim, Hyun-Il Gil, Jae-Hoon Ko, Sun Hye Shin, Byung Woo Jhun, Siew Hoon Sim, Yae-Jean Kim, Louis Yi Ann Chai

**Affiliations:** 1 Division of Infectious Diseases, Department of Medicine, National University Health System, Singapore, Singapore; 2 Department of Pediatrics, Samsung Medical Center, Sungkyunkwan University, Seoul, Republic of Korea; 3 Defence Medical and Environmental Research Institute, DSO National Laboratories, Singapore, Singapore; 4 Department of Pediatrics, Khoo Teck Puat – National University Children’s Medical Institute, Singapore, Singapore; 5 Department of Infectious Diseases, Singapore General Hospital, Singapore, Singapore; 6 Department of Medicine, Yong Loo Lin School of Medicine, National University of Singapore, Singapore, Singapore; 7 Division of Rheumatology, Department of Medicine, National University Health System, Singapore, Singapore; 8 Department of Medicine, Changi General Hospital, Singapore, Singapore; 9 Division of Pulmonary and Critical Care Medicine, Department of Internal Medicine, Kangbuk Samsung Hospital, Sungkyunkwan University School of Medicine, Seoul, Republic of Korea; 10 Division of Infectious Diseases, Department of Medicine, Samsung Medical Center, Sungkyunkwan University School of Medicine, Seoul, Republic of Korea; 11 Division of Pulmonary and Critical Care Medicine, Department of Medicine, Samsung Medical Center, Sungkyunkwan University School of Medicine, Seoul, Republic of Korea

**Keywords:** interferon-alpha, interleukin-12, interleukin-23, *Mycobacterium*, thymic tumor, virus

## Abstract

**Objectives:**

Increased susceptibility to infections is observed in patients with thymomas. These have been invariably attributed to Good syndrome with hypogammaglobulinemia, but there is noticeable heterogeneity in clinical presentation. We clinically and immunophenotypically characterized the infective susceptibilities encountered in these patients.

**Methods:**

Of thymoma patients recruited from Singapore and South Korea, their infection types were correlated against immunological parameters, including IgG, IgM, IgA, CD19+ B cells, and CD4+ T cells, and the presence of neutralizing anti-cytokine autoantibodies using direct ELISA. Lymphocyte subset immunophenotyping was performed. Ascertainment of immune signaling pathway disruption was through serum switch experiments. Respective immune signal outputs were probed using Western blotting.

**Results:**

A total of 15 thymoma patients (median age, 54 years; 13 men [87%]) were clustered into two groups with discernible differences in infective manifestations. In one group, nine patients (60%) had recurrent/severe viral or *Pneumocystis jirovecii* infections. These patients had low immunoglobulins and CD19+ B cells. The second group of six patients (40%) had difficult-to-treat non-tuberculous mycobacterium (NTM) or invasive bacterial or fungal infections. They had normal immunoglobulins levels and possessed autoantibodies against interleukin (IL-)-12, IL--23, or interferon-alpha (IFN-α), which consisted of anti-IFN-α2 and anti-IFN-ω subtypes. The autoantibodies consisted of a heterogeneous spread across IgG1 to IgG4 subclasses. These anti-IL--12, anti-IL--23, and anti-IFN-α autoantibodies were neutralizing and compromised various phosphorylated-STAT signaling pathways that are critical in host anti-pathogen response.

**Conclusions:**

The dichotomy of infective manifestations (viral/PJP versus NTM/invasive bacteria/fungal) underlies distinct and novel immune susceptibility beyond the classic Good syndrome label in thymoma patients, with implications for different approaches to clinical management.

## Introduction

The occurrence of a myriad of infections in patients with thymoma or post-thymectomy observed to date has been loosely attributed to Good syndrome, synonymous with thymoma with hypogammaglobulinemia (1–3). However, more recent descriptions of recurrent or severe infections have been reported in thymoma patients who may not have hypogammaglobulinemia (4). Aberrant parathymic immunological manifestations, including autoimmune propensities with autoantibody generation, are described in these patients, though the etiology is not well understood (5). Here we seek to characterize the varied anti-cytokine autoantibodies and ascertain how they might disrupt immune recognition and signaling pathways to elucidate their roles in the causation of opportunistic infections.

## Methods

Thymoma patients with recurrent severe infections were recruited from medical centers in Singapore and South Korea. Informed consent from each participant was received in accordance with the guidelines of the local ethics committees of the participating institutions. The patients’ clinical information, including age at enrollment, sex, thymoma type, thymectomy history, previous infection histories, autoimmune manifestations, initial immunoglobulin levels (before IVIG replacement if possible), and lymphocyte subset data, were retrospectively reviewed via electronic medical recording systems. Serum samples were collected for anti-cytokine autoantibody screening. The samples were centrifuged and frozen at -70 °C to -80°C within the first 24 h after collection.

Neutralizing autoantibodies against cytokines (IL-6, granulocyte-macrophage colony-stimulating factor (GM-CSF), TNF-α, IL-17, interferon-gamma (IFN-γ), IFN-α, IL-12, and IL-23) were detected using a direct ELISA platform. Confirmation of specific immune signaling pathway disruption was through serum switch experiment using the sera of patients and healthy controls in the presence of control peripheral blood mononuclear cells (PBMC). Immune signal outputs were probed using Western blot. To characterize the autoantibodies, phenotyping into immunoglobulin class and respective sub-classes was performed.

Next, 96-well immunoassay plates (Thermo Fisher Scientific, Waltham, MA, USA) were coated with the respective human recombinant cytokines IL-6, GM-CSF, TNFα, IL-17, IFN-γ, IFN-α, IL-12, and IL-23 (0.5 µg/mL; R&D Systems, Minneapolis, MN, USA) in bicarbonate coating buffer (50 mM, pH 9.6) overnight. The plates were washed with PBS with 0.05% Tween 20 (Sigma-Aldrich, St. Louis, MO, USA), blocked using PBS-T with 5% nonfat dry milk (Bio-Rad, Hercules, CA, USA) for 1 h at room temperature, and washed again. Plasma or serum from each subject was diluted 1,000× in blocking buffer and added to each well. The samples were incubated for 2 h at room temperature, and thereafter the plate was washed. Goat anti-human Fc-specific IgG conjugated with horseradish peroxidase (Nordic MUbio, Susteren, The Netherlands) was added to the plate and incubated for 1 h in the dark. The plates were washed again, and TMB substrate solution (Thermo Fisher Scientific, Waltham, MA, USA) was added to the plate. The reaction was stopped after 30 min with the addition of 2 N H_2_SO_4_ solution, and the plate was measured at 450 nm using BioTek Synergy H1 Plate Reader (Agilent, Santa Clara, CA, USA).

### Serum switch experiments and Western blot analysis

Peripheral blood mononuclear cells (PBMC) isolated from healthy volunteers were treated with sera (10% v/v in RPMI) from either the volunteers or representative thymoma patients who each presented with their respective autoantibodies and then left unstimulated or stimulated with 20 ng/mL rhIL--12 (R&D Systems, USA), 20 ng/mL rhIL--23 (R&D Systems, USA), or 200 ng/mL rhIFN--α2b (Miltenyi Biotec, Germany) at 37 °C for 30 min. The PBMCs were subsequently pelleted and lysed using RIPA buffer, and the protein concentration of the lysates was quantified. Equal amounts of protein from the lysates were loaded and run on 10% SDS–polyacrylamide gels, transferred to PVDF membrane, and probed with antibodies against phosho-STAT1, phospho-STAT3, phospho-STAT4, or anti-TYK2 (Cell Signaling Technology, USA) for analysis. β-actin was the loading control.

### Detection of specific autoantibody subtypes

Nunc Maxisorp 96-well flat-bottomed plates (Thermo Fisher Scientific, MA, USA) were coated with 50 μL per well of 2 μg/mL recombinant human IL--12p70 (Thermo Fisher Scientific, MA, USA), IL--23 (Thermo Fisher Scientific, MA, USA), interferon alpha-2 (Abcam PLC, Cambridge, UK), or interferon omega (Sigma Aldrich, MO, USA) and incubated at 4 °C overnight. After three times of washing with phosphate-buffered saline with 0.05% Tween 20 (PBST), non-specific binding sites were blocked for 1 h at room temperature with BlockerTM Casein (Thermo Fisher Scientific, MA, USA). Following that, the wells were incubated with 1:50 dilution of plasma samples from patients or controls for 2 h, washed with PBST, and incubated with horseradish peroxidase conjugated goat anti-human IgG, IgM (Abcam PLC, Cambridge, UK), IgA (Genomax Technologies, Singapore), and IgE (Thermo Fisher Scientific, MA, USA) for 1 h. After washing, ABTS substrate solution (Sigma Aldrich, MO, USA) was added, and the reaction was stopped with the addition of 1% SDS. The absorbance reading at OD450nm was measured. For IgG-positive samples, the IgG subclasses were determined with 1:10 dilution of plasma samples from patients, with horseradish peroxidase conjugated mouse anti-human IgG1, IgG3 (Thermo Fisher Scientific, MA, USA), IgG2, or IgG4 (Abcam PLC, Cambridge, UK) as secondary antibodies.

## Results

Of the 15 patients with underlying thymoma and being seen for infections (**Table 1**), the majority (80%) had undergone thymectomy at the time of writing. Men predominate (13 out of 15, 86.7%), and the median age was 54.3 (range 41–69) years old.

**Table 1:** Clinical characteristics of patients, including infection types and immunological indices.

Patients	Age	Sex	Primary Infection	Secondary illness	Autoimmune or other non-infectious manifestations	Thymoma WHO subtype	Thymectomy	AutoAb	IgG g/L [5.4- 18.2]	IgA g/L [0.6- 4.8]	IgM g/L [0.2- 2.4]	CD19 cells/uL [65- 250]	CD4 cells/uL [500-1500]	Outcome (cause of death)
I	58	M	Prolonged COVID-19^*^	PML	Nil	AB	Yes (2023)	Negative	3.9	0.2	<0.1	<5	217	Demised (PML)
II	49	M	Prolonged COVID-19^*^	PJP, CMV pneumonitis^#^	Nil	A	Yes (2022)	Negative	1.86	0.05	0.05	<5	436	Alive, on IVIg replacement
III	52	F	CMV end-organ disease^#^ with pneumonitis and retinitis	Infective bronchiectasis exacerbation	Nil	AB	Yes (2016)	Negative	1.1	0.1	0.1	<5	147	Demised (pneumonia)
IV	60	M	CMV retinitis^#^	COVID-19^^^ pneumonia, onychomycosis	Nil	B2	Yes (2013)	Negative	3.05	0.254	0.32	<5	97	Alive, on IVIg replacement
V	45	M	PJP	Enterovirus gastroenteritis	Nil	AB	Yes (2021)	Negative	2.7	0.1	0.1	<5	102	Alive, on IVIg replacement
VI	61	M	Prolonged COVID-19^*^	CMV viremia^#^	Nil	AB	Yes (2023)	Negative	4.76	0.99	0.05	5	390	Alive, on IVIg replacement
VII	42	M	Prolonged COVID-19^*^	Nil	Nil	AB	Yes (2017)	Negative	8.2	1.5	0.1	<5	183	Alive
VIII	69	F	Prolonged COVID-19^*^	Nil	Breast and thyroid papillary carcinoma	C	No	Anti-IFNα, anti- IL23 (light)	7.5	0.8	0.3	<5	1630	Demised (Lost to follow up)
IX	55	M	CMV retinitis	Prolonged COVID-19^*^ pneumonia, HSV stomatitis	Pure red cell aplasia	C	No	Anti-IL12 Anti-IFNα	3.89	0.42	0.17	<5	303	Demised (severe aplastic anaemia, recurrent infection)
X	61	M	*K. pneumoniae, M. abscessus*	Oral candidiasis, COVID-19^^^	MDA5-ILD, Lichen planus,	B	Yes (2020)	Anti-IL12, Anti-IL23	10.6	4.3	1.9	811	694	Demised (myocardial infarction)
XI	53	M	*K. pneumoniae, M. abscessus*, invasive aspergillosis	Onychomycosis, COVID-19^^^	MG, alopecia universalis, vitiligo	AB	Yes (2000)	Anti-IFNα, Anti-IL12, Anti-IL23	17.7	2	1.2	81	64	Demised (pneumonia)
XII	42	M	*M. avium* bronchiectasis	Esophageal candidiasis, VZV infection, COVID-19^^^	MG, alopecia	N.A.	Yes (2012)	Anti-IL12, Anti-IL23	16.63	3.65	1.3	23	259	Lost to follow up
XIII	66	M	Pulmonary *M. abscessus*	Pulmonary aspergillosis and trichosporonosis	Pan-bronchiolitis Pan-sinusitis	C	No	Anti-IL12, Anti-IL23	18.4	1.85	0.49	NA	468	Demised (advanced oligo-metastatic thymoma)
XIV	41	M	Pulmonary melioidosis caused by *Burkholderia pseudomallei*	Nil	Stage 2 chronic kidney disease	AB	Yes (2021)	Anti-IL12, Anti-IL23	11.7	2.8	1.5	169	588	Alive
XV	59	M	*M. abscessus, M. chelonae* bronchiectasis	*Penicillium spp.* pneumonia	Vitiligo	B1, B2	Yes (2015)	Anti-IFNα	19	2.18	1.22	10	92	Demised (unknown)

PJP, Pneumocystis jirovecii pneumonia; CMV, Cytomegalovirus; K. pneumoniae Klebsiella pneumoniae; M. abscessus, Mycobacterium abscessus; M. avium, Mycobacterium avium; M. chelonae, Mycobacterium chelonae; MDA5-ILD, interstitial lung disease with anti-Melanoma Differentiation-Associated gene 5 antibodies; MG, myasthenia gravis; VZV, varicella zoster virus.

^*^ Prolonged COVID-19 infection: non-resolving fever or respiratory symptoms for more than 2 weeks after acute COVID-19 episode, with persistence of SARS-CoV-2 PCR positivity and absence of seroconversion of the anti-SARS-CoV-2 nucleocapsid (N) antibodies at 2 weeks or after (except in Patients II and IX whereby anti-SARS-CoV-2 N antibodies were not measured).

^^^ Patients were diagnosed with acute COVID infection when they had respiratory symptoms and fever together with positive SARS-CoV-2 PCR test.

^#^ CMV infection was confirmed by positive PCR test. CMV reactivation was presumed given that CMV seroprevalence was ~ 90% in the age range of the patients in our populations (14, 15).

Seven patients (I to VII) had low (or borderline low normal range of) IgG levels and hyperglobulinemia of IgM and IgA, which are in keeping with Good syndrome (GS). Susceptibility to viral infections was the key presenting characteristic of this group of patients with low immunoglobulin levels. Refractory SARS-CoV-2 viremia or prolonged COVID-19 lung disease with delayed conversion of anti-SARS-CoV-2 nucleocapsid (N) antibodies were manifestations arising from the COVID-19 pandemic, followed by *Cytomegalovirus* and *Enterovirus* infections and *Pneumocystis jirovecii* pneumonia (PJP). These seven patients with GS did not have anti-cytokine autoantibodies. Two other patients (VIII and IX) with primary presentations of viral infections had low or borderline immunoglobulin levels (of IgG, IgM, or IgA), of which patient IX had detectable autoantibodies against IL--12 and IFN-α (**Table 1**).

We could distinguish a separate patient cohort (X to XV) with distinct infective and immunologic features. These had normal or elevated immunoglobulin levels but presented with recurrent bacterial infections primarily by intracellular pathogens such as non-tuberculous *Mycobacterium* (NTM), *Klebsiella pneumoniae*, and *Burkholderia pseudomallei* or invasive fungal infections. Several patients (X–XII) in this latter cohort also had COVID-19 infection but were able to sero-convert, attaining anti-SARS-CoV-2 N antibody positivity within 2 weeks of acute infection. All of the patients in this group bore autoantibodies against IL--12, IL--23, and/or IFN-α. They did not have autoantibodies against other cytokines, including IFN-γ, IL--17A, IL--6, TNF-α, and GM-CSF. Patients in this latter cohort (X to XV) had immune-mediated co-morbidities such as myasthenia gravis, vitiligo, alopecia universalis, or inflammatory lung disease. It is acknowledged, nonetheless, that patient IX who displayed viral susceptibilities and fulfilled the criteria for GS had pure red cell aplasia and autoantibodies against IL-12 and IFN-α.

**Figure 1:**
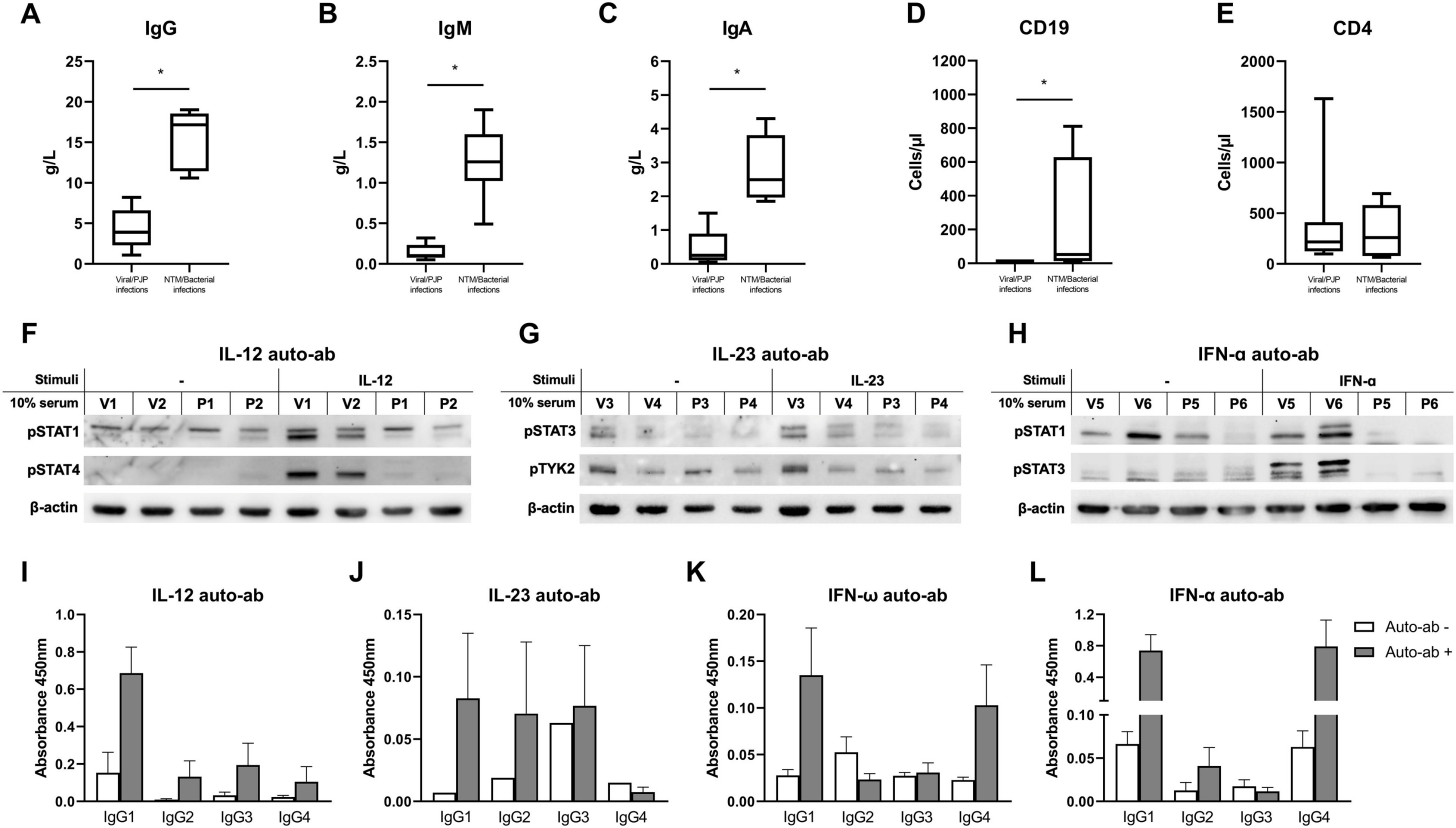
Autoantibody profiles associated with patient groups. **(A–E)** Serum levels of IgG, IgM, and IgA and expression levels of CD19 and CD4 in patients with viral/PJP infections versus NTM/bacterial infections. Data are presented as median and range. *p < 0.05 between infection groups using Mann–Whitney *U*-test. **(F–H)** Western blot showing the neutralizing capacity of 10% sera from respective autoantibody-containing patients (prefix “P”) or healthy volunteers (prefix “V”) against exogenous IL-12, IL-23, IFN-α, or unstimulated (-) in control PBMC. The outputs measured were pSTAT1, pSTAT4, pSTAT3, and pTYK2, respectively, as detected on Western blot with beta(β)-actin as control. **(I–L)** Stratification of anti-IL-12, anti-IL-23, anti-IFN-ω, and anti-IFN-α2 autoantibodies (shaded bars) into IgG1 to IgG4 subtypes against subjects with negative autoantibodies (clear bars). Absorbance reading at optical density of 450 nm (OD450nm) was measured, and data are presented as mean ± SEM. PJP, *Pneumocystis jirovecii* pneumonia; NTM, non-tuberculous *Mycobacterium*.

The patients presenting with primarily viral infections had low IgG (median 3.90 g/L, IQR 2.28–6.62), IgM (median 0.10 g/L, interquartile range 0.08–0.32), and IgA (median 0.25 g/L, IQR 0.10–0.89) (**Figures 1A–C**) as those with GS or the ‘“overlap” borderline-low immunoglobulin group. On the other hand, thymoma patients with NTM or invasive bacterial infections had higher IgG (median 17.17 g/L, IQR 11.43–18.55), IgM (median 1.26 g/L, IQR 1.02–1.60), and IgA (median 2.49 g/L, IQR 1.96–3.81) (*p* = 0.0004). Notably, subjects I to IX (GS and overlap group) had low CD19 (median 5 cells/µL, IQR 5–5) compared to patients (X–XV) who had NTM/bacterial infections and anti-cytokine autoantibodies (median 52 cells/µL, IQR 13–628, *p* = 0.0014) (**Figure 1D**). The mean CD4 counts, however, did not differ between patients (I to IX) in the group with severe/recurrent viral/PJP infection (median 217 cells/µL, IQR 124–413) versus those (X to XV) with anti-cytokine autoantibodies and invasive NTM/bacterial infections (median 259 cells/µL, IQR 78–581, *p* = 0.90) (**Figure 1E**).

We next determined how the detected neutralizing anti-cytokine autoantibodies disrupted the immune signaling of critical host responses against pathogens. STAT1, STAT3, and Tyk2 are known critical signaling mediators of host response against NTM and fungal species (6). The sera of patients with anti-IL-12 autoantibody interfered with control PBMC’s response to IL-12, resulting in attenuated phosphorylation of STAT1 (pSTAT1) and STAT4 (pSTAT4) (**Figure 1F**). Similarly, anti-IL-23 autoantibody-containing sera inhibited the IL-23-triggered signaling of pSTAT3 and Tyk2 (**Figure 1G**); anti-IFN-α autoantibody-containing sera compromised the pSTAT1 and pSTAT3 effector response to IFN--α (**Figure 1H**).

All anti-IFN-α, anti-IL-12, and anti-IL-23 autoantibodies were of IgG type. The anti-IFN-α autoantibody consisted of anti-IFN-α2 and anti-IFN-ω of the 12 known anti-IFN-α isotypes. These antibodies comprised a heterogenous spread of IgG subclasses across IgG1 to IgG4 (**Figures 1I–L**). A comparative inclusion of GS patients as internal controls affirmed that GS patients did not possess these autoantibodies and their respective isotypes.

## Discussion

We elicit here two discernable cohorts of patients with thymoma displaying distinct infective predisposition pointing to differing etiologies for their underlying immune susceptibility. One group was vulnerable to viral infections as exemplified by severe/recurrent CMV and COVID-19, as well as PJP infections, and having low CD19 and immunoglobulin levels: these are the thymoma patients conventionally diagnosed as Good syndrome. Another cohort susceptible to invasive infections by intracellular bacteria pathogens such as *Klebsiella*, NTM, and *Burkholderia* and fungi (*Aspergillus* and *Candida*) had normal or high normal immunoglobulin levels but possessed autoantibodies against IL-12, IL-23, and/or IFN-α. This distinct group of patients ought not to be presumptively labeled or managed as Good syndrome until the appropriate immunological investigative parameters are ascertained.

A few patients with overlap characteristics were encountered, bearing borderline low levels of immunoglobulin and potentially detectable neutralizing anti-cytokine autoantibodies, as exemplified by subject IX who also had red cell aplasia. Akin to GS patients, they present with recurrent/severe viral infections and ought to be considered and managed as for GS, pointing to the utmost importance of immunoglobulins, particularly IgG/IgM, in conferring protection against viruses. It is not known if the presence of anti-cytokine autoantibodies in these few cases might be distinct to the rare and more aggressive thymoma type C.

Notably, all GS patients and those in the “overlap” group had low absolute CD19 counts. This highlights using CD19 count as an alternative but reliable indicative immunological parameter on top of the traditional hypogammaglobulinemia defining GS and alluding to the infective susceptibilities. Having said that, we cannot exclude the role of B cell dysfunction predisposing to infection susceptibility in the latter non-GS cohort (despite having higher CD19 counts and immunoglobulin levels) in the absence of the capacity to ascertain a post-vaccination antibody response. We acknowledge this as a limitation of the current study and pointing to an area of further exploration of this patient cohort at the next opportunity.

What we have advanced here is the means to clinically delineate and identify thymoma patients who are Good syndrome against those most likely to carry these anti-IL-12, anti-IL-23, or anti-IFN-α autoantibodies. We consolidate this further by ascertaining how these autoantibodies affect the respective host signaling and effector immune responses, including through pSTAT1, pSTAT3, and Tyk2, during infection with bacteria, *Mycobacteria*, and fungi (7). These autoantibodies are heterogenous across the range of IgG1–IgG4 subclasses. Despite predisposition to autoantibody generation, these patients notably do not produce autoantibodies against IFN-γ as seen in another unique cohort of patients for which the exact etiologies for both are still not fully ascertained (8). Neither do these patients generate detectable amounts of autoantibodies against IL-17A, IL-6, TNF-α, and GM-CSF as we have tested (data not shown). A hypothesis to account for this phenomenon is that IL-12 and IL-23 (which share a common p40 subunit) and IFN-α are known to be co-expressed intrinsically by human thymocytes and thymic plasmacytoid dendritic cells (pDC) (9, 10), upon which autoimmunization may occur (11). This can be facilitated by defective negative selection induced by aberrant auto-reactive naïve T cell subpopulations through the loss of peripheral tissue-specific antigens (TSAgs) in the medullary thymic epithelial cells (mTECs), resulting in the escape of inherent checks which maintain immune tolerance (12). Conversely in GS, it is perceived that aberrant T cell precursors induce broad-based antigen-directed B (and T) cell subset lymphocytopenia, which consequently manifests as hypogammaglobulinemia (13).

It remains to be ascertained if patients with thymoma-related anti-cytokine autoantibodies could have increased disease and mortality burden due to the autoimmune-linked co-morbidities as our data seem to point to, contributed by the demonstrated interference of immune-sensing by the autoantibodies and the chronicity of NTM infections. This study is limited by the small patient number, and recruitment had been at the point of presentation with opportunistic infections, whereas ideally patients would have been prospectively immunophenotyped and followed up from the point of diagnosis of thymoma. However, our study stands out in recognizing the existence of two groups of distinct infective susceptibilities in thymoma patients and that traditional Good syndrome diagnosis might have been an over-simplification. This differentiation, based on infection type and simplified immunologic parameters in thymoma, enables the identification of the probable mechanistic disease process, which is important by the bedside as treatment may be different. Immunoglobulin replacement is the treatment for Good syndrome, whereas definitive antimicrobial treatment remains the first-line treatment for bacterial and fungal infections in patients with anti-cytokine autoantibodies. Plasmapheresis may be resorted to in the setting of critical infection unresponsive to antimicrobial therapy in the presence of high autoantibody titer, as roles for B cell therapy and adjunctive immunomodulatory treatment, including steroids or other agents, have yet to be ascertained.

## Data Availability

The original contributions presented in the study are included in the article/supplementary material. Further inquiries can be directed to the corresponding author.
